# Syndrome de Behçet associé à une néphropathie à Ig A

**DOI:** 10.11604/pamj.2015.20.410.5956

**Published:** 2015-04-27

**Authors:** Madiha Mahfoudhi, Rym Goucha

**Affiliations:** 1 Service de Médecine Interne A, Hôpital Charles Nicolle, Tunis, Tunisie

**Keywords:** Maladie de Behçet, hématurie, néphropathie à Ig A

## Image en medicine

Le syndrome de Behçet est une vascularite qui peut s'accompagner de plusieurs atteintes systémiques. Une atteinte rénale peut compliquer cette maladie. Les lésions rénales sont variées, les plus fréquentes sont l'amylose et la néphropathie à IgA. Patient âgé de 33 ans suivi pour un syndrome de Behçet depuis 10 ans et dont le diagnostic a été retenu devant l'association d'une aphtose bipolaire récidivante, des lésions de pseudofolliculite et un test pathergique positif. Il avait un typage HLA B 51. L'examen ophtalmologique était normal. Il a présenté une hématurie microscopique. L'examen cytobactériologique des urines était négatif. L’évolution était marquée par la survenue d'une hématurie macroscopique, un syndrome néphrotique avec une protéinurie à 3,8 g/24h et une albuminémie à 25 g/l ; la fonction rénale était normale. Il n'avait pas de lithiases urinaires. L'uréthro-cystographie rétrograde, l'uro-scanner et l’échographie doppler rénale étaient sans anomalies. Une origine lithiasique, infectieuse ou tumorale a été éliminée. Le dosage des IgA était élevé. La ponction biopsie rénale a objectivé une néphropathie glomérulaire avec des dépôts mésangiaux diffus d'IgA . Un traitement par l'azthioprine et la colchicine a été instauré. Le patient n'a pas présenté de récidive de l'hématurie sur un recul de 2 ans avec disparition du syndrome néphrotique. La réalisation d'analyses urinaires systématiques à la recherche d'une hématurie et/ou d'une protéinurie au cours d'un syndrome de Behçet permet de faire le diagnostic précoce d'une néphropathie à Ig A.

**Figure 1:**
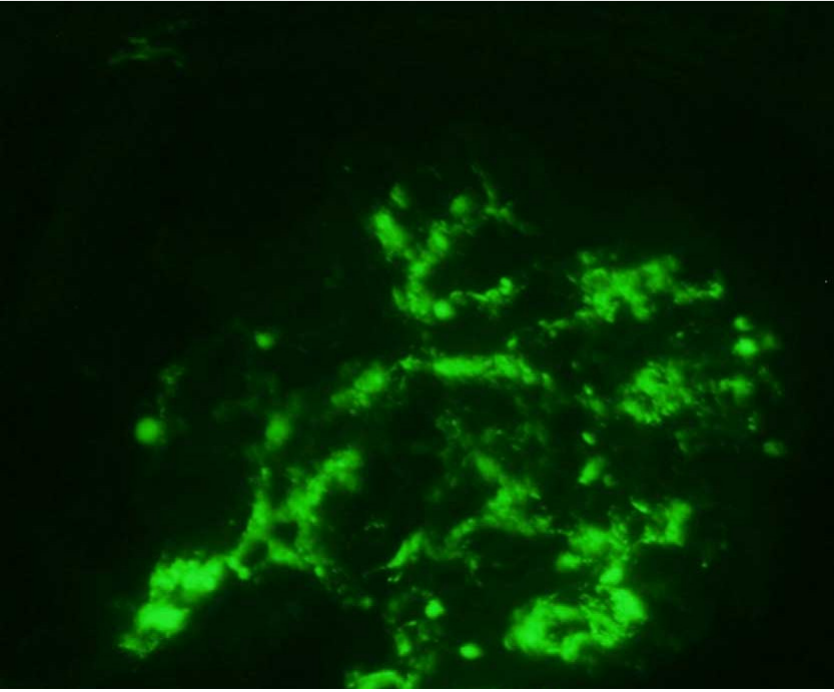
Immunofluorescence directe avec un anticorps anti-IgA couplé à la fluorescéine: un glomérule présentant des dépôts d'IgA de siège mésangial diffus

